# Identification and Characterization of ZEL-H16 as a Novel Agonist of the Histamine H_3_ Receptor

**DOI:** 10.1371/journal.pone.0042185

**Published:** 2012-08-01

**Authors:** Ying Shi, Rong Sheng, Tingting Zhong, Yu Xu, Xiaopan Chen, Dong Yang, Yi Sun, Fenyan Yang, Yongzhou Hu, Naiming Zhou

**Affiliations:** 1 College of Life Sciences, Zhejiang University, Hangzhou, Zhejiang Province, China; 2 College of Pharmaceutical Sciences, Zhejiang University, Hangzhou, Zhejiang Province, China; Universidad Federal de Santa Catarina, Brazil

## Abstract

The histamine H3 receptor (H3R) has been recognized as a promising target for the treatment of various central and peripheral nervous system diseases. In this study, a non-imidazole compound, ZEL-H16, was identified as a novel histamine H3 receptor agonist. ZEL-H16 was found to bind to human H3R with a *Ki* value of approximately 2.07 nM and 4.36 nM to rat H3R. Further characterization indicated that ZEL-H16 behaved as a partial agonist on the inhibition of forskolin-stimulated cAMP accumulation (the efficacy was 60% of that of histamine) and activation of ERK1/2 signaling (the efficacy was 50% of that of histamine) at H3 receptors, but acted as a full agonist just like histamin in the guinea-pig ileum contraction assay. These effects were blocked by pertussis toxin and H3 receptor specific antagonist thioperamide. ZEL-H16 showed no agonist or antagonist activities at the cloned human histamine H1, H2, and H4 receptors and other biogenic amine GPCRs in the CRE-driven reporter assay. Furthermore, our present data demonstrated that treatment of ZEL-H16 resulted in intensive H3 receptor internalization and delayed recycling to the cell surface as compared to that of control with treatment of histamine. Thus, ZEL-H16 is a novel and potent nonimidazole agonist of H3R, which might serve as a pharmacological tool for future investigations or as possible therapeutic agent of H3R.

## Introduction

Histamine, a biogenic amine with multiple physiological effects, exerts its biological activities through four distinct G-protein-coupled receptors (GPCRs) known as the histamine H1, H2, H3, and H4 receptors [1], [2]. The histamine H1 and H2 receptors were identified decades ago [3], [4], and have been shown to be excellent drug targets for the treatment of allergy and gastric ulcers, respectively [5], [6]. The histamine H4 receptor was discovered in 2000 [7], and it has been identified as a potential target for the treatment of inflammatory diseases such as chronic allergies, asthma, atopic dermatitis, and inflammatory bowel diseases [8]. The histamine H3 receptor (H3R) was first identified by Arrang and colleagues in 1983 using a functional assay in which it was found that histamine inhibits its own synthesis and release [9]. However, the cloning of the human histamine H3 receptor cDNA in 1999 by Lovenberg and colleagues [10] prompted H3R research in both academia and industry.

Although H3R mRNA is detectable in the heart, lung, gastrointestinal tract, and endothelial cells [11], H3R is predominantly expressed in the central nervous system (CNS) and peripheral nervous system. The highest levels of H3R are found in the cerebral cortex, hippocampal formation, basal ganglia, and hypothalamus [12], [13]. It has been established that H3R associates with the heterotrimeric Gi/o-protein, which leads to a decrease in cAMP formation and PKA activation, and also causes the activation of the Akt/GSK-3β axis and ERK1/2 pathways, the inhibition of the Na^+^/H^+^ exchanger, and modulation of intracellular calcium upon histamine stimulation [14]. The H3R was first identified as a presynaptic autoreceptor that negatively regulated the synthesis and release of histamine from histaminergic neurons [9]. However, the H3R has also been shown to act as a presynaptic heteroreceptor on non-histaminergic neurons, inhibiting the release of other neurotransmitters such as acetylcholine, dopamine, norepinephrine, serotonin, and various neuropeptides in both the central and peripheral nervous system [15], [16], [17]. Therefore, the H3 receptor has long been recognized as a promising target for the treatment of various central and peripheral nervous system diseases. Antagonists and inverse agonists of the H3 receptor have been proposed as potential drugs for the treatment of attention-deficit hyperactivity disorders (ADHD), Alzheimer’s disease, obesity and others [18], [19], [20], [21], whereas H3R agonists are suggested for the treatment of asthma, migraine, and ischemic arrhythmias. However, increasing evidence suggests H3R agonists could serve as potential therapeutics for obesity, diabetes mellitus, and liver cholestasis [22], [23], [24]. BP 2–94, one of the earliest explored agonists of H3R, displayed anti-inflammatory and anti-nociceptive properties in mice and was recognized as a promising drug for the treatment of asthma, migraines, related inflammatory diseases, and pain [25], [26]. N-α-methylhistamine, another promising agonist of H3R, was tested in a Phase III clinical study for migraine prophylaxis [23]. Therefore, it is reasonable to believe that H3R agonists could hold therapeutic value for the treatment of human diseases.

In the current study, ZEL-H16 was identified as a novel agonist of H3R using CRE-luciferase assay and internalization assay with HEK-293 cells stably expressing H3R. Affinities of ZEL-H16 for hH3R and rH3R were measured by competition binding experiments. We also investigated the ability of ZEL-H16 to induce the phosphorylation of ERK1/2 in mouse cortical neuronal cultures expressing endogenous H3R and the ability to inhibit the contraction of the guinea-pig ileum. Another major result of this study is that ZEL-H16 could induce intense internalization and delay recycling of internalized H3R to the cell surface compared with histamine. Our results clearly demonstrate that ZEL-H16 is a potent, selective and non-imidazole agonist of H3R that could serve as a useful pharmacological tool for future studies or as a possible therapeutic agent.

## Results

### Characterization of ZEL-H16 as a Selective Partial H3R Agonist in CRE Reporter Assay

**Figure 1:**
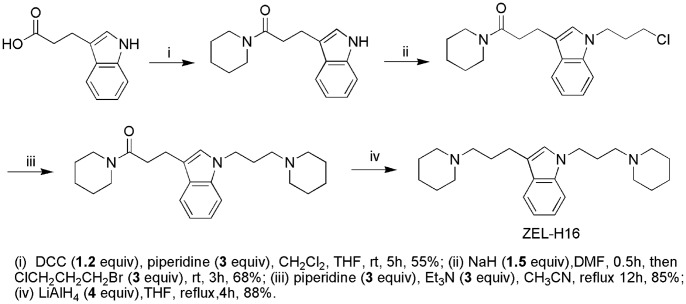
Chemical structure and synthetic routes for ZEL-H16. Reaction of indole-3-propionic acid with piperidine provided 2, which was alkylated with 1-bromo-3-chloropropane in the presence of NaH in anhydrous DMF to get 3. Reaction of 3 with piperdine in refluxing acetonitrile afforded 4, followed by reduction with LiAlH4 to yield ZEL-H16.

**Figure 2:**
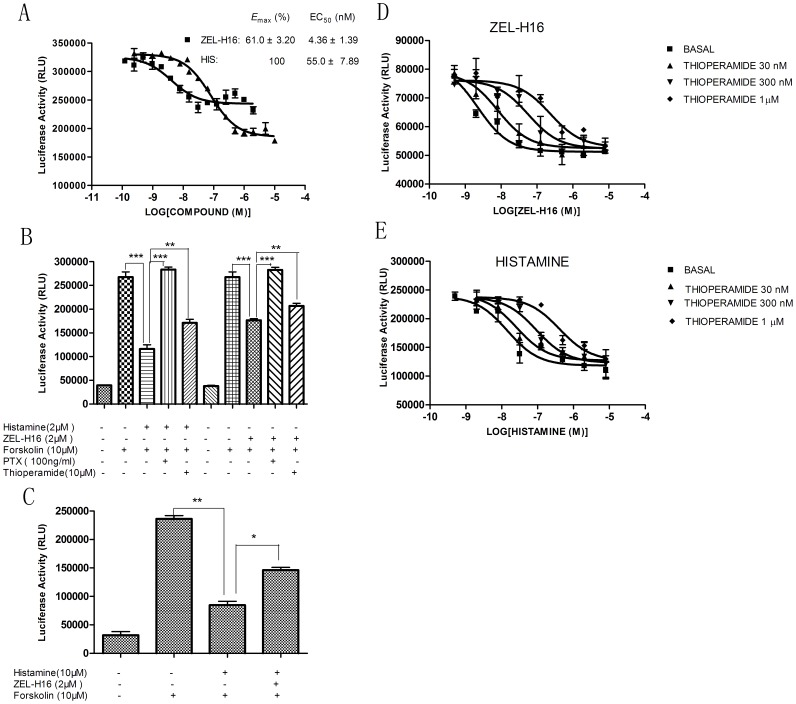
Identify ZEL-H16 as a partial agonist in CRE-driven luciferase activity assay in HEK-293 cells stably expressing hH3R. A, Concentration inhibition curve of luciferase activity induced by ZEL-H16 with forskolin stimulation. B, Luciferase activity with Forskolin stimulation in response to ZEL-H16 and histamine in the absence and presence of PTX or Thioperamide. C. Luciferase activity with Forskolin stimulation in response to 10 µM Histamine in the presence of 2 µM ZEL-H16. D and E. Luciferase activity with Forskolin stimulation in response to ZEL-H16 (D) and histamine (E) in the absence and presence of 30 nM, 300 nM or 1 µM Thioperamide. The presented data points are the mean ±SE of triplicate values from a single experiment and are representative of three to six separate experiments (*p<0.05; **p<0.01).

We used CRE-driven reporter assay as primary assay to screen more than 300 antagonist compounds in 6 serials of structures, and found several compounds with the activity in inhibition of forskolin-induced luciferase activity. We then employed hH3R-GFP redistribution assay as a secondary assay to confirm the antagonistic activity. One of the compounds identified, ZEL-H16, whose chemical structure and synthetic routes are shown in Figure 1, triggered a significant increase in receptor internalization as compared to the control compound histamine, behaving as an agonist on H3R internalization. The agonist activity of ZEL-H16 was further confirmed in HEK-293 cell lines that stably express the human H3R and a reporter gene consisting of the firefly luciferase coding region that is under the control of minimal promoter containing cAMP-response elements (CREs). The CRE-driven reporter assay is widely used to measure the function of GPCR agonists and antagonists. Changes of intracellular cAMP could cause changes of CRE-driven report gene transcription. As indicated in Figure 2A, compound ZEL-H16 has partial agonistic properties and a low EC_50_ value for H3R as compared to histamine (EC_50[ZEL-H16]_  = 4.36±1.39 nM, *E*_max[ZEL-H16]_  = 61.0±3.20%, Mean ± SEM, n = 6; EC_50[histamine]_  = 55.0±7.89 nM, *E*_max[histamine]_  = 100%, Mean ± SEM, n = 6). The agonistic activity of ZEL-H16 could be disrupted by co-incubation with PTX, a Gi inhibitor, and thioperamide, an antagonist of H3R (Fig. 2B). Moreover, the inhibition of forskolin-induced luciferase activity by Histamine could be reduced by co-incubation with ZEL-H16 (Fig. 2C). We also performed the experiments to determine the CRE-driven luciferase activity in the response to ZEL-H16 and histamine in the presence of three different concentrations of antagonist thioperamide (Fig. 2D and 2E), and obtained the Schild slopes 1.116±0.256 for histamine, 1.140±0.168 for ZEL-H16, that were not significantly different from unity. The data suggested that it is likely for both ZEL-H16 and histamine to bind to the same binding site of H3 receptor.

**Figure 3:**
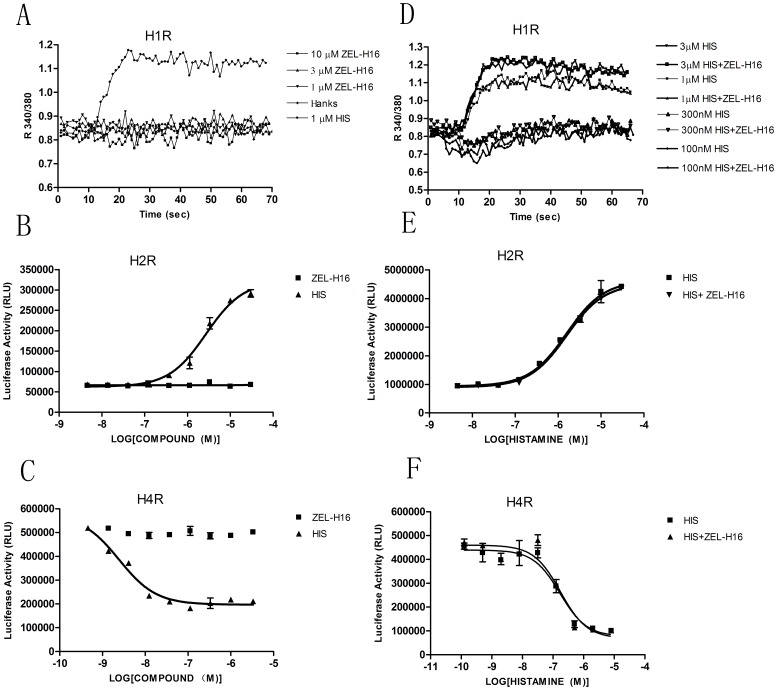
The selectivity of ZEL-H16 for hH3R in intracellular Ca^2+^ flux assay and CRE-luciferase transcription assay. A–C, Intracellular Ca^2+^ flux or CRE-driven luciferase activity induced by ZEL-H16 in HEK-293 cells stably expressing hH1R (A), hH2R (B), or hH4R (C). D–F, The influence of ZEL-H16 on intracellular Ca^2+^ flux or CRE-driven luciferase activity induced by histamine in HEK-293 cells stably expressing hH1R(D), hH2R(E), or hH4R(F). The presented data points are the mean ±SE of triplicate values from a single experiment and are representative of three separate experiments. Statistical analysis between ZEL-H16 added or not added for each concentration point in the kinetic graph was done using a t-test (PRISM software). G, Relative expression of hH1R, hH2R, hH3R and hH4R on transfected HEK-293 cells by ELISA quantification for Flag-tagged cell-surface receptors (***p<0.001, compared to non-transfected HEK 293 cells).

We then examined the selectivity of ZEL-H16 versus various histamine receptors by assaying intracellular Ca^2+^ flux and cAMP formation. As shown in Fig. 3A and 3D, ZEL-H16 did not induce similar intracellular Ca^2+^ release in H1R-expressing HEK-293 cells compared to histamine, neither modulate the intracellular Ca^2+^ release stimulated by histamine. In addition, no significant responses were observed in the luciferase activity after stimulation by ZEL-H16 in cells expressing H2R or H4R (Fig. 3B and 3C). The addition of ZEL-H16 also did not significantly change the luciferase activity curves induced by histamine in H2R or H4R- expressing cells (Fig. 3E and 3F). The relative expression of H1R, H2R, H3R and H4R on transfected cell membrane was shown in Figure 3G. The selectivity of ZEL-H16 toward other biogenic amine GPCRs, such as dopamine receptor DRD1 and DRD2, serotonin receptor 5-HT_1A_, adrenergic receptor α_2_AR was also examined by CRE-luciferase activity assay. ZEL-H16 showed no agonist or antagonist activities to these receptors in the experiments (Table 1). These results suggested that ZEL-H16 is a potent and selective H3 receptor agonist.

**Table 1:** The selectivity of ZEL-H16 on other biogenic amine GPCRs in CRE-luciferase transcription assays^a^.

	Agonist Activity	ZEL-H16	
Receptors	Agonist EC_50_(nM)	Agonist Activity	Antagonist Activity
DRD1	dopamine 51.6±17.1	–	–
DRD2	dopamine 90.7±11.3	–	–
5-HT_1A_	serotonin 33.6±10.3	–	–
α_2_AR	epinephrine 4.30±2.52	–	–

a HEK-293 cells transiently expressing receptors were treated with different concentration of agonist with or without 2 µM ZEL-H16 or different concentration of ZEL-H16 separately. ZEL-H16 had no significant influence on cAMP formation alone and neither on cAMP formation induced by their corresponding agonists in the CRE- luciferase activity assays. Statistical analysis between ZEL-H16 added or not added for each concentration point was done using a t-test (PRISM software).

### Direct Binding of ZEL-H16 to Human H3R and Rat H3R

**Figure 4:**
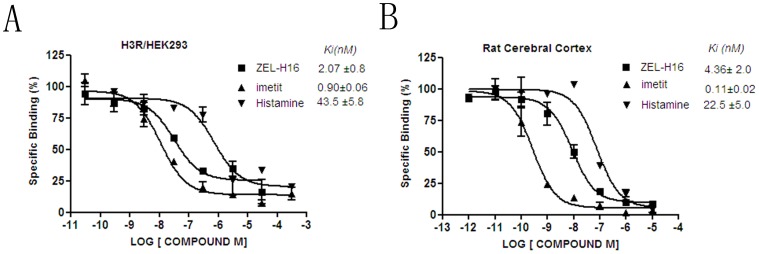
Competition binding experiments with [^3^H]-N-α-methylhistamine. A. H3R/HEK-293 cell membranes with 10 nM [^3^H]-N-α-methylhistamine; B. Rat cerebral cortex membranes with 2 nM [^3^H]-N-α-methylhistamine. The presented data are the means ±SE of values from a single experiment performed in duplicate and are representative of three to six separate experiments.

Competitive binding experiments were conducted to assess whether ZEL-H16 could directly bind to H3R. Scatchard analysis of the saturation binding using [^3^H]N-α-methylhistamine against human histamine H3 receptor revealed a K_D_ value of 0.71 nM and a Bmax value of 430 fmol/mg protein (data not shown). In the competitive binding assays, binding of [^3^H]N-α-methylhistamine to human H3R expressing HEK-293 cells was blocked by cold histamine, ZEL-H16 and imetit, yielding 43.5±5.8, 2.07±0.8 and 0.9±0.06 nM of the *Ki* values respectively (Fig. 4A), and binding of [^3^H]N-α-methylhistamine to rat cerebral cortex was also inhibited by cold histamine, ZEL-H16 and imetit, yielding 22.5±5.0, 4.36±2.0 and 0.11±0.02 nM of the *Ki* values respectively (B_max_ = 767 fmol/mg protein, K_D_ = 0.830 nM) (Fig. 4B). The results showed that ZEL-H16 could directly bind to hH3R and rH3R.

### Internalization of H3R Induced by ZEL-H16 in HEK-293 cells Stably Expressing H3R

**Figure 5:**
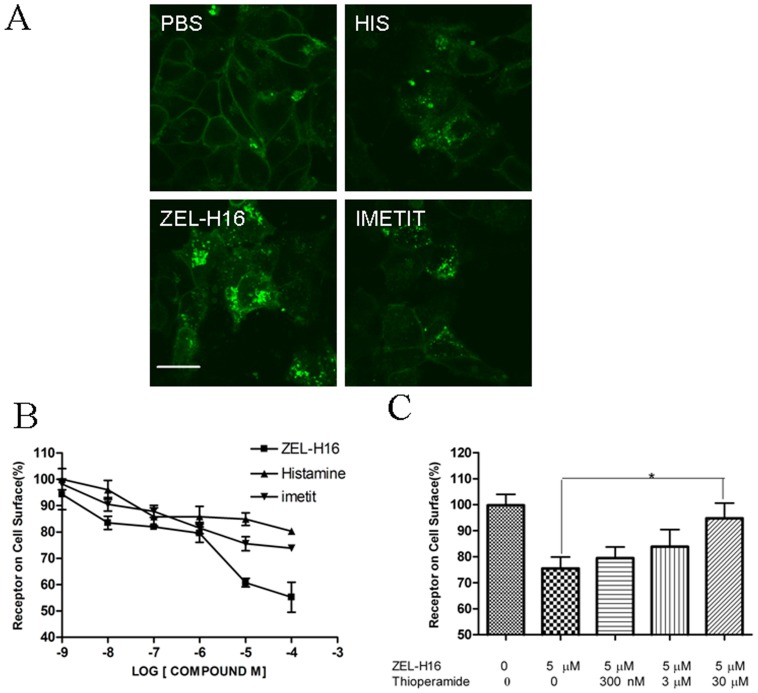
Internalization of H3R-EGFP stably expressed in HEK-293 cells induced by ZEL-H16. A, HEK-293 cells stably expressing H3R-EGFP were stimulated with histamine, ZEL-H16, or imetit for 45 min respectively. (a) control; (b) 100 µM Histamine; (c) 100 µM ZEL-H16; (d) 100 µM imetit. B, ELISA quantification for Flag-tagged cell-surface receptors showed a concentration -dependent internalization of H3R induced by ZEL-H16. C, Internalization of H3R induced by 5 µM ZEL-H16 in the absence and presence of thioperamide (*p<0.05). ELISA data are expressed as the percentage of receptors detected on the surface of agonist-untreated cells expressing H3R. Error bars represent the SEM for four replicates.

Next, we conducted internalization experiments to determine the ability of ZEL-H16 to induce H3R internalization. HEK-293 cells stably transfected with H3R-EGFP were incubated with 100 µM ZEL-H16, histamine and imetit for 45 min at 37°C separately and internalization was examined by confocal microscopy. As seen in Figure 5A (c), H3R internalized from the cell surface into the cytoplasm with a punctuate distribution upon activation by ZEL-H16. Moreover, the receptor internalization induced by ZEL-H16 was more intense than the internalization induced by histamine and imetit (Fig. 5A). This result was confirmed by experiments that quantified the levels of receptors on the surface of the cells following stimulation by compounds. HEK-293 cells stably transfected with Flag-H3R were treated with different concentrations of ZEL-H16 or histamine or imetit for 45 min at 37°C respectively, and the amount of H3R remaining on the cell surface was quantitatively detected by ELISA. Quantification by cell-surface ELISA showed a significant loss of cell-surface receptors due to treatment with ZEL-H16 at concentration ranging from 1–100 µM (Fig. 5B). Treatment with 100 µM ZEL-H16 caused a 50% loss in the expression of the receptors on the cell surface, whereas the same concentration of histamine or imetit only induced approximately 18–20% internalization of cell-surface receptors. This result suggested that ZEL-H16 may be a powerful tool to investigate the internalization behavior of H3R. In addition, H3R internalization induced by 5 µM ZEL-H16 could be blocked by pre-incubation with thioperamide for 20 min at high concentration (Fig. 5C).

**Figure 6:**
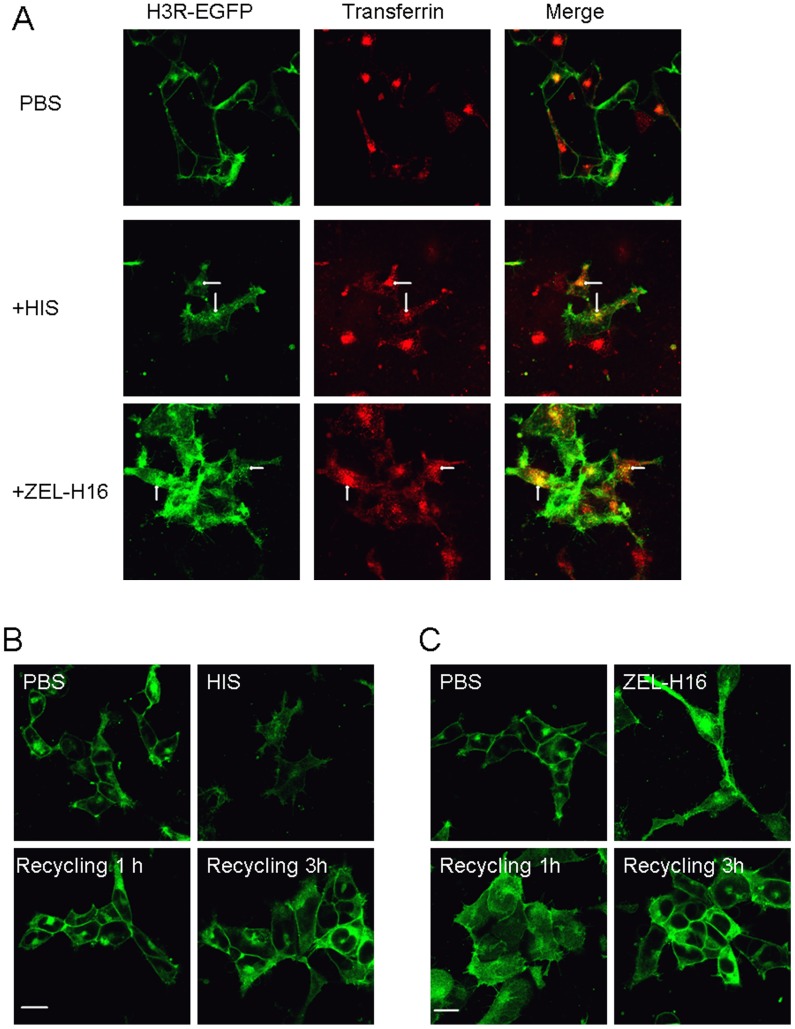
Localization of internalized H3R-EGFP stably expressed in HEK-293 cells and recycling of internalized H3R to the cell surface. A. HEK-293 cells stably expressing H3R-EGFP were incubated with or without 20 µM ZEL-H16 and 500 µM histamine in the presence of 100 g/ml Alexa Fluor546-labeled transferrin for 45 min at 37°C. B. H3R-EGFP expressing cells were treated with 100 µg/ml cycloheximide and 20 µM ZEL-H16 or 500 µM histamine at 37°C for 30 min, followed by the removal of residual agonists by washing, and further incubation in the presence of cycloheximide for the indicated time periods. The internalized receptors were recycled to the plasma membrane within 1 h after histamine removal and 3 h after ZEL-H16 removal. All pictures shown are representative of at least three independent experiments.

Furthermore, we used the endosome marker Alexa Fluor 546-labeled transferrin to assess whether internalized H3Rs induced by histamine or ZEL-H16 are generally recycled back to the plasma membrane via early and recycling endosomes. Confocal microscopy analysis revealed that the internalized H3R receptors induced by histamine or ZEL-H16 were both colocalized with transferrin in endosomes (Fig. 6A). The recycling experiments showed that the internalized H3R receptors were recycled to the cell surface within 1 h after histamine removal, but within 3 h after ZEL-H16 removal (Fig. 6B and C), suggesting that internalized H3Rs with ZEL-H16 exhibited delayed recycling to the cell surface compared with histamine.

### ZEL-H16-induced Activation of ERK1/2 in HEK-293 Cells Stably Expressing H3R

**Figure 7:**
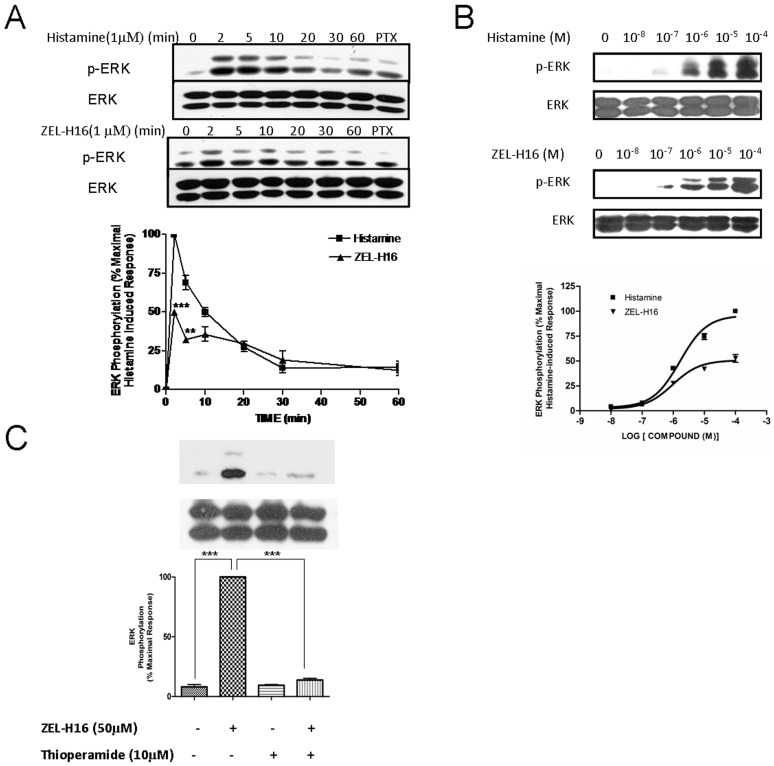
Phosphorylation of ERK1/2 induced by ZEL-H16 in H3R-expressing HEK-293 cells. A, time-dependent phosphorylation of ERK1/2 induced by histamine or ZEL-H16. Following 3 h of serum starvation, cells were treated with 1 µM histamine or ZEL-H16 for the indicated time period and cell lysates were analyzed for phosphorylated ERK (p-ERK) and total ERK (ERK) protein levels. Signals were quantified by densitometric image analysis and p-ERK was normalized to a loading control (ERK). The signal at each point is expressed as the percentage of the maximal p-ERK signal induced by histamine. A statistical analysis between ZEL-H16 and histamine for each time point in the kinetic graph was done using a t-test (PRISM software) (*p<0.05; **p<0.01). B, Concentration-dependent phosphorylation of ERK1/2 induced by histamine or ZEL-H16. After serum starvation, cells were incubated with increasing concentrations of ZEL-H16 or histamine (10 nM to 100 µM) and cell lysates were analyzed for p-ERK and ERK levels. Concentration -dependent phosphorylation signals were quantified by densitometric analysis and p-ERK levels were normalized to total ERK levels. The signal at each point is expressed as the percentage of the maximal p-ERK signal induced by histamine. C, The phosphorylation of ERK1/2 induced by ZEL-H16 could be entirely abolished by co-incubation with H3R antagonist thioperamide (***p<0.001). Data represent the mean ±SE of three independent experiments.

We next examined ERK1/2 phosphorylation induced by ZEL-H16 and histamine to determine their signal transduction function in HEK-293 cells stably expressing H3R. Cells were incubated with 1 µM ZEL-H16 or 1 µM histamine (Fig. 7A) for several time points (0–60 min) and the phosphorylation of ERK1/2 was assessed by Western-blotting analysis. The results revealed that maximal ERK1/2 activation occurred 2 min after stimulation with ZEL-H16 or histamine. The maximal activation of ERK1/2 achieved with ZEL-H16 was approximately 50% of the maximal activation of ERK1/2 induced by histamine. The kinetic graph suggested that ZEL-H16 has partial activation properties against H3 receptors in the activation of MAPK (Fig. 7A).

To determine if ERK1/2 activation occurs in a concentration-dependent manner, cells were incubated with increasing concentrations of ZEL-H16 or histamine (10 nM to 100 µM) (Fig. 7B). Western-blot analysis showed that ZEL-H16 induced ERK1/2 phosphorylation in a concentration-dependent manner and has an EC_50_ of 1.1±0.1 µM. This EC_50_ value was similar to the EC_50_ induced by histamine, 1.6±0.1 µM.

We also investigated the effect of PTX on the phosphorylation of ERK1/2 induced by ZEL-H16 by pre-incubating the cells with 100 ng/mL PTX for 12 h. As shown in Fig. 7A (lane 8), PTX completely inhibited ERK1/2 activation induced by both ZEL-H16 and histamine. Co-incubation of ZEL-H16 with 10 µM thioperamide, an H3R antagonist, also completely abolished the stimulatory effects of 50 µM ZEL-H16 (Fig. 7C). Taken together, these data suggested that stimulation with ZEL-H16 elicited transient phosphorylation of ERK1/2 via H3 receptors through a PTX sensitive Gi/o signaling pathway.

### ZEL-H16-induced Activation of ERK1/2 in Neonatal Mouse Cortical Neurons

**Figure 8:**
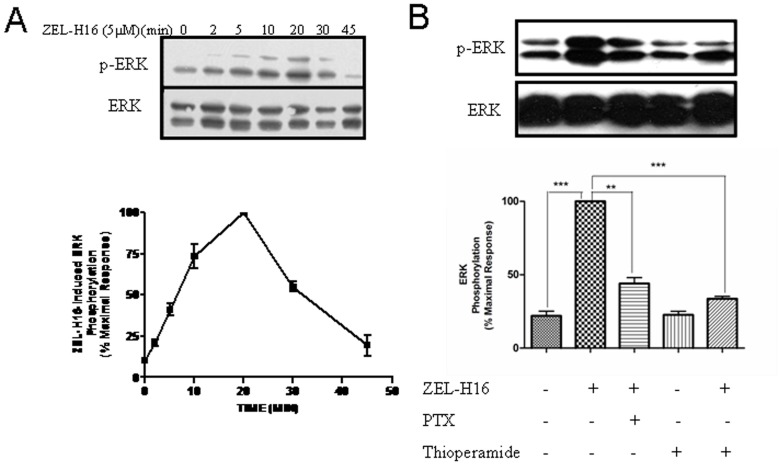
Phosphorylation of ERK1/2 induced by ZEL-H16 in mouse cortical neurons. A, Time-dependent phosphorylation of ERK1/2 induced by 5 µM ZEL-H16 in neonatal mouse cortical neuron cultures. B, The phosphorylation of ERK1/2 induced by 50 µM ZEL-H16 for 20 min was mostly abolished by 100 ng/mL PTX or 10 µM thioperamide. Signals were quantified by densitometric image analysis and p-ERK was normalized to a loading control (ERK). The signal at each point is expressed as the percentage of the maximal p-ERK signal induced by ZEL-H16. Data represent the mean ±SE of three independent experiments.

The mouse H3 receptor protein is 94.1% identical to the human H3 receptor protein. Therefore, we also examined the effect of ZEL-H16 on ERK phosphorylation in H3R endogenously expressing mouse cortical neurons. Neonatal mouse cortical neuron cultures were treated with ZEL-H16 after seven days of primary culture and analyzed by specific anti-phospho-ERK immunoblots. H3R transcription was previously detected by RT-PCR in these cultures (Fig. S1). Treatment with 5 µM ZEL-H16 produced a time-dependent and significant change in ERK phosphorylation in these neuronal primary cultures (Fig. 8A). The maximal activation of ERK occurred 20 min after stimulation with ZEL-H16, which lagged behind the maximal response induced in HEK-293 cells expressing H3R. Co-incubation with 10 µM thioperamide or 100 ng/ml PTX could abolish most of the stimulatory effects induced by 50 µM ZEL-H16 at 20 min (Fig. 8B). These data suggested that ZEL-H16 could also function on mouse H3R and activate the MAPK signal pathway in mouse cortical neurons.

### Guinea-pig Ileum Bioassay

**Figure 9:**
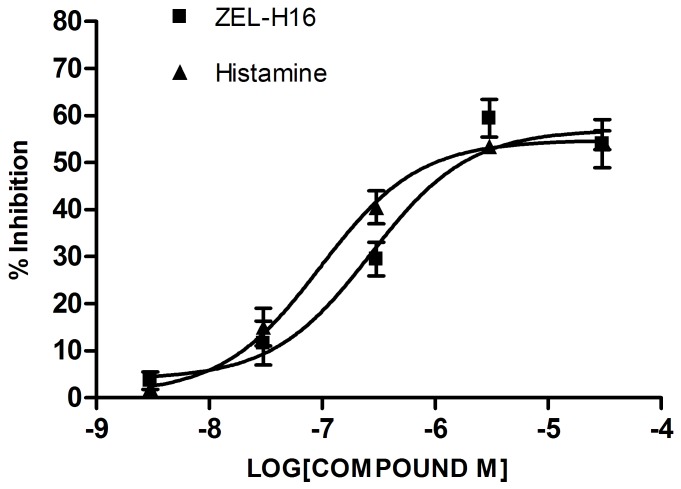
Effect of ZEL-H16-induced and histamine-induced inhibition of the electrically induced contraction of the guinea-pig ileum. Each point is the mean ± s.e.m. of three to six separate experiments.

We next used Guinea-pig ileum bioassay as an in vitro model to assess the activity of ZEL-H16. In Guinea-pig ileum bioassay, ZEL-H16 produced concentration–dependent inhibition of the electrically induced twitch of the guinea-pig ileum just like Histamine (Fig. 9). Both ZEL-H16 and Histamine inhibit the contractions of the guinea pig ileum with a comparable efficacy (54.03±5.13% and 54.52±0.99%, respectively) and potency (p*D*_2_ 6.57±0.23 and 7.02±0.15, respectively). This result indicated that ZEL-H16 behaved as a full agonist of H3R in inhibition of the electrically induced twitch of the guinea-pig ileum just like Histamine, although ZEL-H16 was identified as a partial agonist of H3R in CRE-driven reporter assay and in ERK1/2 phosphorylation.

## Discussion

The histamine H3 receptor, which is predominantly expressed in the CNS, has been known to modulate the release of various neurotransmitters including histamine, dopamine [27], [28], acetylcholine [29], [30], norepinephrine [31], serotonin [32], [33], GABA [34], glutamate [35], and substance P [36]. Therefore, H3R has been identified as a potential therapeutic target for many nervous system diseases. Classical agonists of the histamine H3 receptor, e.g., (R)-a-methylhistamine [37], [38], imetit [39] and Immepip [40], consist of the imidazole ring as a common structural feature. The discovery of the histamne H4 receptor evoked strong interest of many pharmaceutical companies to develop H4R selective ligands for the regulation of immune functions with possible uses in allergy and asthma [41]. The alignment of amino acid sequences of the human histamine H4 receptors with H3 receptor suggested an overall 43% identity homology [42]. Consequently, many imidazole-based histamine H3 receptor agonists also possess a high affinity at the H4 receptor. As one of the most potent and selective H3 agonist, immepip also shows reasonable potency for the histamine H4 receptor with pKi values of 7.66 [43]. In the current study, we have identified a new non-imidazole based H3 agonist, ZEL-H16, which exhibited approximately 20-fold higher affinity for human H3R as compared to histamine. ZEL-H16 showed no agonist or antagonist activities at the other histamine receptors H1, H2, and H4 in the CRE-luciferase assay (Figure 3), suggesting that ZEL-H16 exhibits high selectivity among histamine H1, H2, H3 and receptors and other biogenic amine GPCRs including serotonin receptor, α2 adrenergic receptor and dopamine receptors. In addition, ZEL-H16 also exhibited a characteristic feature of induction of intense H3 receptor internalization and delayed recycling to the cell surface as compared to that of control with treatment of histamine. Thus, ZEL-H16 is identified as a potent and highly selective histamine H3 receptor agonist, which will be useful as a tool in H3R research.

Further characterization of ZEL-H16 using CRE-driven luciferase assay and ERK1/2 phosphorylation assay demonstrated that ZEL-H16 acted as partial agonist in inhibition of forskolin-induced cAMP formation (the efficacy was 60% of that of histamine) and activation of ERK1/2 signaling pathway (the efficacy was 50% of that of histamine). Both of ZEL-H16-mediated inhibition of forskolin-induced cAMP production and ERK1/2 phosphrylation were PTX-sensitive, suggesting the involvement of Gi protein in cAMP accumulation and ERK1/2 phosphorylation. However, in Guinea-pig ileum bioassay, ZEL-H16 exhibited concentration–dependent inhibition of the electrically induced twitch of the guinea-pig ileum as a full agonist just like histamine. Previous studies have demonstrated that depending on the test model, iodoproxyfan and FUB 407 have been described as antagonists, partial or even full agonists [44], [45]. GT-2331 and proxyfan acted as full agonists in cAMP assays, but they displayed weak partial agonism in modulating neurotransmitter release examination [46]. Betahistine has been found to act as a nanomolar inverse agonist and a micromolar agonist at histamine H3 receptors on in vitro inhibition of cAMP formation and [^3^H]arachidonic acid release, but to behave as a partial inverse agonist on in vivo measuring tele-methylhistamine levels in the brains of mice [47]. These differences in pharmacological action in different assays might be caused by a varying receptor reserve or species variants of the histamine H3-receptor protein as well as by different experimental conditions within the assays [48].

It is well established that rapid internalization of the agonist-activated receptor into the intracellular membrane compartments of target cell plays an important role in the regulation of GPCR signaling and desensitization [49]. To visualize the internalization and trafficking of human histamine H3 receptors, we constructed a vector to express a chimeric protein by fusing enhanced green fluorescent protein (EGFP) to the C terminal end of the H3 receptor (H3R-GFP). In stably H3R-GFP-expressing HEK-293 cells, H3R-EGFP was mainly localized at the plasma membrane and was rapidly internalized in a concentration- and time- dependent manner upon agonist stimulation. ZEL-H16 treatment at the concentration of 100 µM led to a 50% loss of cell surface expression, whereas exposure of cells to the same concentration of histamine and imetit induced an 18–20% loss of H3R from cell surface. Moreover, our observation using immunofluorescence indicated that the internalized H3 receptors induced by both ZEL-H16 and histamine were co-localized with early endosome containing the transferrin receptors. When histamine was removed, the internalized H3 receptors were recycled to the cell surface within one hour; however, the receptors internalized in the presence of ZEL-H16 were recycled to the cell surface until 3 hours after wash-out of compound, consistent with the obervation of amiooxypentane-RANTES (AOP-RANTES)-mediated human CCR5 internalization [50]. Further study has demonstrated that the internalized CCR5 molecules mediated by AOP-RANTES do recycled to the cell surface with kinetics equivalent to those of receptors in RANTES-exposed cells. However, these recycled CCR5 receptors are rapidly reinternalized [51]. The mechanism of slower recycling of internalized H3 receptors in the presence of ZEL-H16 remains for further elucidation.

In conclusion, a new non-imidazole compound, ZEL-H16, was identified as a novel and selective agonist of histamine H3 receptor. Our present data showed that ZEL-H16 selectively bind to H3 receptors, leading to PTX-sensitive inhibition of forskolin-induced intracellular cAMP formation and activation of ERK1/2. It is interesting to note that exposure of cells to ZEL-H16 resulted in intensive H3 receptor internalization and delayed recycling to the cell surface as compared to control compound histamine. As a novel agonist, ZEL-H16 and its derivatives might serve as useful pharmacological tool for future investigations regarding the molecular and pharmacological aspects of H3R or as a possible therapeutic agent for the treatment of human diseases.

## Materials and Methods

### Ethics Statement

All animal work was conducted in accordance with the Guide for the Care and Use of Laboratory Animals as adopted and promulgated by the United States National Institutes of Health. The protocol was approved by the research ethics committee of Zhejiang University (approval ID: Zju2010-1-01-020).

### Drugs and Materials

Forskolin, PTX, histamine, imetit, thioperamide, dopamine, serotonin and epinephrine were obtained from Sigma-Aldrich (St. Louis, MO). DMEM medium and fetal bovine serum were purchased from Hyclone (Beijing, China). Lipofectamine 2000 and G418 were obtained from Invitrogen (Carlsbad, CA). The pEGFP-N1 and pCMV-Flag vectors were purchased from Clontech Laboratories, Inc. (Palo Alto, CA) and Sigma (St. Louis, MO), respectively. Primary antibodies for p-ERK1/2 and total ERK were purchased from Cell Signaling (Danvers, MA). [^3^H] N-α-methylhistamine (NET-1027∶83 Ci/mmol) was purchased from Perkin-Elmer Life Sciences. Analysis data of ZEL-H16: ^1^H NMR (500 MHz, CDCl_3_): δ 7.59 (d, 1 H, *J = *8.0 Hz, H-4), 7.33 (d, 1 H, *J = *8.0 Hz, H-7), 7.19 (t, 1 H, *J = *7.0 Hz, H-6), 7.08 (t, 1 H, *J = *8.0 Hz, H-5), 6.89 (s, 1 H, H-2), 4.14 (t, 2 H, *J = *7.0 Hz, -N_1_CH_2_CH_2_CH_2_N-), 2.76 (t, 2 H, *J = *7.5 Hz, -C_3_CH_2_CH_2_CH_2_N-), 2.42−2.36 (m, 8 H, piperidine-2,6), 2.33−2.30 (m, 2 H, -N_1_CH_2_CH_2_CH_2_N), 2.26 (t, 2 H, J = 7.0 Hz, -C_3_CH_2_CH_2_CH_2_N-), 1.99−1.97 (m, 2 H, -N_1_CH_2_CH_2_CH_2_N-), 1.94−1.87 (m, 2 H, -C_3_CH_2_CH_2_CH_2_N-), 1.61−1.56 (m, 8 H, piperidine-3,5), 1.45−1.42 (m, 2 H, piperdine-4); ESI-MS: m/z  = 368.2 [M+H]^+^; IR (KBr): ν 2930, 2859, 1611, 1583, 1459, 1360, 730cm^-1^; HPLC purity 99.38%.

### Constructs

The hH1R and hH2R genes were cloned from HEK-293 genomic DNA by PCR. The hH3R gene was cloned using human thalamus poly-A RNA (Clontech, Palo Alto, CA) with RT-PCR methods. The hH4R gene was cloned from human bone marrow Marathon-Ready cDNAs (Clontech, Basingstoke, U.K.) by PCR. Primers were designed according to the published human histamine receptor gene sequences (GenBank accession no. X76786, M64799, AF140538, and AB044934). The expression vectors of human DRD1, DRD2, and 5-HT_1A_ were provided by Dr. Jinpeng Sun (the School of Medicine of Shandong University), and the expression vector of human α2AR was purchased from GenScript USA Inc.(Nanjing, Jiangsu). All cDNAs were sequenced and separately cloned into the pCMV-Flag and pEGFP-N1 expression vectors.

### Cell Culture and Generation of Stable Cell Lines

HEK-293 cells were cultured in Dulbecco’s modified Eagle’s medium (DMEM) supplemented with 10% fetal bovine serum, 100 U/mL penicillin, and 100 µg/mL streptomycin in a humidified atmosphere of 95% air and 5% CO_2_ at 37°C. For transfection, H3R cDNA plasmid constructs were transfected or co-transfected into HEK-293 cells using Lipofectamine 2000 according to the manufacturer’s instructions. Stable transfectants were selected in the presence of 800 µg/mL G418.

### Membrane Preparation

HEK-293 cells stably expressing H3R were harvested and resuspended in ice-cold 50 mM Tris-HCl (pH 7.4) containing a complete protease inhibitor cocktail tablet and mixed by inverting. The cell pellet was homogenized with a Polytron PT 1200 set at 4000 rpm and centrifuged at 1,000 g for 5 min. The supernatant was then centrifuged twice at 50,000 g for 30 min at 4°C. Finally, the resulting pellet was gently suspended in 50 mM Tris-HCl (pH 7.4) buffer and the membrane concentration was determined using Bio-Rad protein assays.

The male rats (180 g–200 g) were killed by decapitation, and the cerebral cortex was rapidly removed in ice-cold 50 mM Tris-HCl (pH 7.4) containing a complete protease inhibitor cocktail tablet. The cerebral cortex was homogenized with a Polytron (at maximum setting, 3×10 sec) and centrifuged twice at 1,000 g at 4°C to separate the nuclear fraction and cell debris. The resulting supernatants were combined and then centrifuged twice at 50,000 g for 20 min at 4°C to obtain tne membrane fraction. Protein concentration was determined using Bio-Rad protein assays.

### Competitive Binding Assays

The membranes (50 µg of protein) were incubated with [^3^H] N-α-methylhistamine at various concentrations (from 0.03 nM to 50 nM) for saturation binding assays. For competitive binding assays, the membranes (50 µg of protein) were incubated with 10 nM [^3^H] N-α-methylhistamine for recombinant hH3R or 2nM [^3^H] N-α-methylhistamine for rat brain H3R in the presence of various compound concentrations in the buffer (50 mM Tris–HCl, pH 7.4). The reaction mixtures were incubated for 90–120 min at room temperature to achieve binding equilibrium. Following three washes, the membrane-bound radioactivity was measured on a Topcount (PerkinElmer) at 20°C. Non-specific binding was determined in the presence of excessive cold histamine (final conc., 300 µM). IC_50_ values were converted to *Ki* values using the Cheng-Prusoff equation. Each experiment was performed in duplicate and repeated three to six times. All data were analyzed by nonline arregression using GraphPad Prism version 4.0 software.

### CRE-driven Reporter Gene Assay

Stable HEK-293 cells co-transfected with H3R and pCRE-Luc were seeded in a 48-well plate overnight and were grown to 90×95% confluence. Next, the cells were stimulated with 10 µM forskolin or 10 µM forskolin plus different concentrations of histamine or compound in serum-free DMEM, and the cells were incubated for 5 h at 37°C. Luciferase activity was detected using a firefly luciferase kit (Promega, Madison, WI). When required, cells were treated overnight with or without PTX (100 ng/ml) in serum-free DMEM before the experiment.

### Intracellular Calcium Measurement

The stably H1R-expressing HEK-293 cells were harvested with Cell Stripper (Mediatech, Herndon, VA), washed twice with phosphate-buffered saline (PBS), and resuspended in Hanks’ balanced salt solution (140 mM NaCl, 5 mM KCl, 10 mM HEPES, pH 7.4, 1 mM CaCl_2_, 1 mM MgCl_2_, 1 mg/ml glucose) containing 0.025% bovine serum albumin. The cells were then loaded with 2 µM Fura-2 acetoxymethyl ester derivative (Fura-2/AM) (Molecular Probes, Eugene, OR) for 30 min at 37°C. Cells were washed once in Hanks’ solution, resuspended in Hanks’, incubated at room temperature for 15 min, washed twice in Hanks’ solution, and then resuspended in Hanks’ at a concentration of 3×10^6^ cells/ml. A typical experiment contained 1.0×10^5^ cells/100 µl in a well of 96-well plate. These cells were stimulated with the indicated concentrations of compounds. HEK-293 cells stimulated with 3 µM histamine or ZEL-H16 were used as a negative control respectively. Calcium flux was measured using excitation at 340 and 380 nm in a Infinite 200 PRO multifunctional microplate reader (Tecan Austria GmbH, Grödig, AT ) and [Ca^2+^]i was measured using the 340/380 excitation ratio (R 340/380).

### Internalization Assay by Confocal Microscopy

HEK-293 cells stably expressing H3R-EGFP were seeded in covered glass-bottom 6-well plates. After 24 h, cells were treated with histamine or ZEL-H16 for the indicated times at 37°C. After fixing the cells with 4% paraformaldehyde for 10 min, cells were mounted in mounting reagent (DTT/PBS/glycerol). Confocal images were taken on a Zeiss LSM 510 microscope with an attached Axiovert 200 microscope and LSM5 computer system. Images were collected using QED camera software and processed with Adobe Photoshop.

### Measurement of Cell Surface Receptors by ELISA

The cell surface expression of H3R was quantitatively assessed by ELISA and performed as described previously [52]. Briefly, HEK-293 cells stably transfected with the pCMV-H3R construct were seeded in 48-well dishes coated with poly-L-lysine. The next day, the cells were stimulated with the indicated concentrations of histamine and ZEL-H16 for the indicated times. Medium was aspirated and the cells were washed once with Tris-buffered saline (TBS). After fixing the cells for 5 min at room temperature with 3.7% formaldehyde in TBS, the cells were washed 3 times with TBS and then blocked for 45 min with 1% bovine serum albumin/TBS. Cells were then incubated for 1 h with an alkaline phosphatase-conjugated monoclonal antibody directed against the Flag epitope and diluted 1∶1,000. Cells were washed 3 times, and antibody binding was visualized by adding 0.25 mL of an alkaline phosphatase substrate (Bio-Rad). Development was stopped by adding 0.1 mL of the substrate to a 96-well microtiter plate containing 0.1 mL of 0.4 M NaOH. Plates were read at 405 nm in a microplate reader (Bio-Rad) using Microplate Manager software.

### Western Blot Analysis

HEK-293 cells stably expressing human H3R were grown in a 6-well plate and were serum starved 2 h in serum-free culture medium prior to stimulation. Cells were stimulated with the indicated H3R ligands at the designated concentrations. Incubations were stopped at the indicated times by removing the stimulation medium by aspiration. The cells were washed in ice-cold PBS and subsequently lysed in 100 µl of lysis buffer [20 mM HEPES (pH 7.5), 10 mM EDTA, 150 mM NaCl, 1% Triton X-100, and one tablet of complete protease inhibitor (Roche, Indianapolis, IN) per 50 mL] at 4°C on a rocker for 30 min. The lysates were centrifuged and separated by sodium dodecyl sulfate-polyacrylamide (12%) gel electrophoresis (SDS-PAGE) and blotted onto polyvinylidene difluoride membranes. Membranes were blocked for 1 h at room temperature in TBST (10 mmol/L Tris, 150 mmol/L NaCl, 0.1% Tween-20, pH 8.0) buffer containing 5% (v/w) skim milk. Membranes were probed overnight with primary antibodies against p-ERK1/2 (1∶1,000; Cell Signaling, Danvers, MA) in TBST containing 5% (v/w) BSA, and then probed with horseradish peroxidase-labeled secondary antibody (1∶5,000) for 1 h at room temperature in TBST containing 5% (v/w) milk powder. Immunoreactivity was detected by ECL assays. The blots were stripped and reprobed using antibodies against total ERK1/2 (1∶2,000) (Cell Signaling, Danvers, MA) as a control.

### Analysis of ERK Phosphorylation in Primary Cultures of Cortical Neurons

Neuronal cortical cultures were prepared from neonatal ICR mice. Cerebral cortices were dissociated in sterile Dulbecco’s phosphate-buffered saline (D-PBS; Sigma-Aldrich, St. Louis, MO), and the neurons were isolated in the same medium containing 0.5% trypsin at 37°C for 10 min. After centrifugation, dissociated neurons were re-suspended in neurobasal medium (NBM; Gibco, Carlsbad, CA) supplemented with 2% B-27 and 0.5 mmol/L-glutamine (Gibco, Carlsbad, CA) and then plated on 48-well plates at a density of approximately 1.3×10^6^ per well. After 7 days of culture, the neurons were serum starved for approximately 3 h prior to drug treatment. After the cells were stimulated by H3R ligands, the neurons were harvested in lysis buffer and proteins were separated using SDS-PAGE and then transferred onto PVDF membranes. Immunoblotting was performed as previously described.

### Guinea-pig Ileum Assay

The procedure used was as described previously [53]. Adult male guinea pigs (300–500 g) were killed by cervical dislocation. The ileum was removed at a point 20 cm from the caecum and flushed with and placed in modified K–H buffer of following composition: 118 mM NaCl, 5.9 mM KCl, 1.2 mM CaCl_2_, 1.2 mM MgSO_4_, 1 mM Na_2_HPO_4_, 25 mM NaHCO_3_ and 10 mM D-glucose. Ileum segments (2.5–3 cm) were suspended in 20 ml organ baths containing K–H buffer maintained at 37°C and gassed with 95% O_2_/5% CO_2_. Contractile activity under stimulation (rectangular pulses of 15 V, 0.5 ms, and 0.1 Hz) was recorded using isometric transducers (Grass FTO3). Concentration–response effects of histamine or ZEL-H16 were obtained in different each tissue. Mepyramine (3 mM) and famotidine (10 mM) (Sigma-Aldrich, St. Louis, MO) were added to the K–H buffer to block postsynaptic H1 and presynaptic H2 receptors, respectively.

### Data Analysis

Sigmoidal agonist concentration-response curves (in the presence and absence of antagonists) were created through computer-assisted nonlinear regression using the GraphPad Prism program (GraphPad Software, San Diego, CA, USA). Schild slopes (n) were constructed from linear regression of the Schild equation as the following:


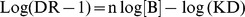


DR is the dose-ratio as the EC50 in the presence of antagonist divided by the EC50 in the absence of antagonist. B is the concentration of the antagonist. These points were then fitted to a straight line. A slope of 1 then indicates competitive antagonism [54]. All data are presented as mean ± SEM. Then in the text refers to the number of separate experiments.

## Supporting Information

Figure S1 A, the 7 th –day’s cortical neurons cultures of neonatal mouse. B, RT-PCR detection of mouse H3R of the 7 th –day’s cortical neurons cultures of neonatal mouse.
(TIF)
