# Correction: Identification and Characterization of ZEL-H16 as a Novel Agonist of the Histamine H_3_ Receptor

**DOI:** 10.1371/annotation/c536426a-169e-4d9a-8382-cdda0378aa29

**Published:** 2012-08-30

**Authors:** Ying Shi, Rong Sheng, Tingting Zhong, Yu Xu, Xiaopan Chen, Dong Yang, Yi Sun, Fenyan Yang, Yongzhou Hu, Naiming Zhou

There were errors in the Funding section. The correct funding information is as follows: This work was supported by grants from the Ministry of Science and Technology (2012CB910402， 2012AA020303-05 and 2008AA02Z138), and the National Key Tech Project for Major Creation of New drugs (NO. 2009ZX09501-003). The funders had no role in study design, data collection and analysis, decision to publish, or preparation of the manuscript. 

