# Supplementary figures and images for: Identification and Characterization of ZEL-H16 as a Novel Agonist of the Histamine H3 Receptor
Source: PLoS One. 2012 Aug 1;7(8):e42185. doi: 10.1371/journal.pone.0042185 (PMC3411647; doi:10.1371/journal.pone.0042185)

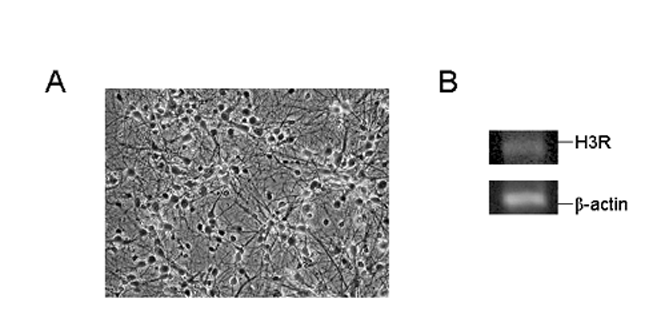

Supplement: Figure S1 — A, the 7 th –day’s cortical neurons cultures of neonatal mouse. B, RT-PCR detection of mouse H3R of the 7 th –day’s cortical neurons cultures of neonatal mouse. (TIF) [file pone.0042185.s001.tif]
